# Idiopathic Inflammatory Myopathies—Treatment Perspective of Highly Specialised Rheumatology Centre

**DOI:** 10.3390/jcm15103658

**Published:** 2026-05-09

**Authors:** Maria Dutsch-Wicherek, Piotr Szczęsny, Małgorzata Wisłowska

**Affiliations:** 1 Faculty of Medicine, Medical University of Warsaw, 02-091 Warsaw, Poland; 2 National Institute of Geriatrics, Rheumatology and Rehabilitation, 02-637 Warsaw, Poland

**Keywords:** idiopathic inflammatory myopathy (IIM), juvenile dermatomyositis (JDM), dermatomyositis (DM), clinically amyopathic dermatomyositis (CADM), inclusion body myositis (IBM), polymyositis (PM), immune-mediated necrotising myopathy (IMNM), anti-synthetase syndrome (ASyS), rituximab, JAK inhibitors, anifrolumab, sirolimus

## Abstract

**Background/Objectives:** Idiopathic inflammatory myopathies (IIMs) are chronic immune-mediated disorders, causing striated muscle weakness and extramuscular symptoms. Real-world, single-centre data are needed to interpret phenotype patterns and evolving therapies. **Methods**: A single-centre, retrospective cohort study was conducted at the Rheumatology Clinic of the National Institute of Geriatrics, Rheumatology and Rehabilitation, Warsaw, Poland from 1 January 2022 to 31 December 2025. Data included demographics, IIM subtypes, extramuscular involvement, co-existing Sjögren disease (SD), biopsy results, autoantibodies, and treatment. Due to sample size, descriptive analysis was used. **Results**: The study included 35 patients (31.4% men). Mean age was 50.7 years; mean body mass index (BMI) was 26.0 kg/m^2^. The cohort consisted of 10 dermatomyositis (DM), one polymyositis (PM), two immune-mediated necrotising myopathy (IMNM), one inclusion body myositis (IBM), 16 anti-synthetase syndrome (ASyS), four juvenile dermatomyositis (JDM), and one clinically amyopathic dermatomyositis (CADM). SD co-occurred in eight cases, including six cases of ASyS. Anti-Jo1 was observed in 13 ASyS cases and one DM. Glucocorticoids (GCSs) were administered in all patients for induction in addition to cyclophosphamide (28.6%), mycophenolate mofetil (MMF) (51.4%), and methotrexate (MTX) (17.1%). Maintenance therapy included MTX (20%), MMF (31.4%), rituximab (34.3%), azathioprine (AZA) (42.9%), and others. Two DM, two JDM, and one ASyS patient received JAK inhibitors, one DM and one JDM anifrolumab, one IBM sirolimus, and four patients with interstitial lung disease (ILD) nintedanib. **Conclusions**: This Polish single-centre cohort shows effective use of novel therapies for IIM. Sirolimus, JAK inhibitors, and nintedanib were effective. Co-occurrence of SD in ASyS patients requires further research.

## 1. Introduction

Idiopathic inflammatory myopathies (IIMs) are a rare, heterogeneous group of immune-mediated disorders with predominant skeletal muscle involvement and frequent multisystem manifestations, including skin, lungs, joints, gastrointestinal tract, and heart [1,2,3]. Proximal muscle weakness and myalgia are characteristic. Annual incidence ranges from 11–660 per 1,000,000 person-years [1].

The classification is currently based on the 2017 EULAR/ACR criteria, which distinguishes subtypes including dermatomyositis (DM), clinically amyopathic DM (CADM), polymyositis (PM), inclusion body myositis (IBM), immune-mediated necrotising myopathy (IMNM), antisynthetase syndrome (ASyS), and juvenile DM (JDM) [4].

The current nomenclature, developed by Gianni et al. [5], includes JDM and other juvenile myositis (JM) in paediatric cases; and in adults, DM (including CADM), PM, IBM, IMNM, antisynthetase syndrome (ASyS), and overlap of myositis (OM) with other connective tissue diseases. Non-specific and cancer-associated myositis (CAM) have also been recognized [1].

PM, DM, and IMNM predominate in women, while IBM is more common in men [1]. Early diagnosis is essential, as organ involvement is strongly associated with prognosis [5].

We summarize the main subtypes of IMNM: DM, CADM, PM, IBM, IMNM, ASyS, and JDM.

### 1.1. Laboratory Examinations

Myositis-specific autoantibodies (MSA) are present in approximately 60% of patients: the most common are anti-Jo-1 antibodies; others include anti-SRP, anti-Mi-2, anti-MDA5, anti-NXP2, and anti-TIF-1γ antibodies [6]. Some MSA antibodies help predict clinical phenotypes and cancer risk. Anti-NXP2, anti-TIF-1, and anti-Mi-2 antibodies are associated with an increased risk of cancer [6].

MSA antibodies strongly correlate with specific clinical features. Anti-Jo-1 antibodies are associated with ILD, mechanics hands, Raynaud’s phenomenon, and arthritis. Antiaminoacyl-tRNA synthetases other than Jo-1 exhibit the same features, except for arthritis, and are strongly associated with periungual erythema. Anti-SRP antibodies correlate with cardiac involvement. Anti-Mi-2 and anti-NXP2 antibodies are associated with skin involvement and dysphagia. Anti-MDA5 antibodies are exclusive to patients with DM and are strongly associated with ILD. Anti-TIF-1γ antibodies are associated with the DM phenotype, periungual erythema, dysphagia, and CAM, particularly in patients aged 58 years and older. Anti-SAE antibodies correlate with skin involvement and periungual erythema in the amyopathic phenotype [6].

Myositis-associated antibodies (MAAs) are detected in patients with other connective tissue diseases. The most common antibodies include anti-PMScl, anti-U1RNP, anti-Ro60, anti-Sm, anti-Ku, anti-La, anti-U3, anti-RNA Pol, anti-Topo, and AMA, which are less common [6]. In 20–30% of patients, IIM is seronegative and has no identifiable autoantibodies [1,6].

Patients with myositis typically have elevated levels of muscle enzymes, including creatine kinase (CK) and aldolase. CK is the most sensitive marker and can be used to monitor disease activity [1]. In 20% of patients with myositis, particularly in patients with JDM or IBM, CK levels may be within the normal range [7]. Aldolase may be the only enzyme elevated in patients with DM [2].

### 1.2. Muscle Involvement

Typical symptoms of myositis include symmetric proximal muscle weakness, myalgia, neck flexor weakness, dysphagia, and dysphonia [1]. Muscle weakness develops subacutely in DM and ASyS, more rapidly in IMNM. Leg muscles are typically weaker than arm muscles, except for the OM [1]. Myalgia is relatively uncommon in patients with IBM. Muscle symptoms develop gradually over the years. Dysphagia occurs in more than half of patients, leading to malnutrition or death [8].

Electromyography (EMG) differentiates the causes of muscle weakness and distinguishes neurogenic from primary muscular causes, and chronic from acute myositis. Typical EMG in 70–80% of patients with IIM shows short, low-amplitude, polyphasic motor unit action potentials and early recruitment patterns [9]. Myositis manifests in MRI most commonly as muscle oedema.

Muscle biopsy is best performed under ultrasound or MRI guidance especially in equivocal or seronegative cases [9,10,11].

The presentation of PM includes CD8+ T cell invasion and surrounding of non-necrotic muscle fibres. DM cases are diagnosed by perivascular and perimysial CD4+ T cell infiltration and perifascicular myofibre atrophy [11]. IMNM is characterised primarily by macrophage infiltrates and necrotic myofibres; cellular infiltrates may be sparse [9]. Biopsy in IBM visualises characteristic rimmed vacuoles, amyloid deposits, and features resembling PM. Rimmed vacuoles are quite characteristic, but they can be absent in 25–67% of cases [1]. Moreover, scarce rimmed vacuoles can be present in PM cases [7]. ASyS muscle pathology demonstrates perimysial and perivascular macrophages, CD8+ and CD4+ T lymphocytes, and even CD20+ B lymphocytes. Necrotic and regenerative fibres can be found perifascicularly [1].

### 1.3. Skin Manifestations

Dermatological signs include Gottron’s sign (a hyperkeratotic skin rash extending mainly over the extensor side of joints with violaceus macules that occur in 60–80% of patients over the fingers, wrists, elbows, knees, and ankles), Gottron’s papules (violaceus, pink or dusky red hyperkeratotic papules, located over the dorsal side of the metacarpal or interphalangeal joints), heliotrope rash (a periorbital violaceus, hyperkeratotic or oedematous erythema of one or both—in 50% of patients), shawl sign (red or violaceous erythema of the neck, shoulders, and chest), calcinosis (calcium hydroxyapatite deposits in the subcutaneous and soft tissue, mechanic’s hands in antisynthetase syndrome patients (scaling and fissuring on the radial side of the index finger and ulnar side of the thumb, often with hyperkeratosis; the equivalent sign in the feet is called hiker’s foot). Generalised oedema and erythema are rare and correlate with anti-TIF-1γ autoantibodies. Calcinosis most commonly occurs in DM; it is also associated with OM and systemic sclerosis [10,11].

### 1.4. Respiratory Tract Involvement

Dyspnoea is common among IIM patients and can result from various forms like ILD, pulmonary arterial hypertension (PAH), and diaphragmatic or respiratory muscle weakness. It is the most frequent extra-muscular manifestation and correlates with worse prognosis, as well as early mortality [12,13]. When diaphragm or respiratory muscle weakness occurs, subclinical respiratory muscle weakness is common, but clinically significant respiratory muscle weakness is rare and may be life-threatening, leading to acute respiratory failure requiring mechanical ventilation. PAH occurs almost exclusively in patients with IIM and ILD.

The highest risk of ILD occurs in certain groups of IIM patients, i.e., in anti-MDA5 DM/CADM and ASyS with anti-Jo1, anti-PL7, or anti-PL12 [11]. Mechanic’s hands and Raynaud phenomenon are associated with ILD. Interestingly, cancer has a negative association with ILD [12,13]. Rapidly progressive ILD (RP-ILD) develops over days to weeks with severe dyspnoea and acute hypoxic failure and is associated with a high mortality rate (40–90%). Anti-Mi-2, anti-TIF-1γ, or anti-NXP2 antibodies correlate with a lower risk of RP-ILD. Chronic ILD (C-ILD) develops gradually over months to years and responds better to treatment than RP-ILD, i.e., 89% of patients respond to GCS monotherapy [12]. In comparison, the 3-month survival of RP-ILD patients treated with GCSs alone is 51% [12]. Patients experience dyspnoea and a productive cough. Subclinical ILD manifests only in radiographic findings and correlates with severe muscular involvement [12].

### 1.5. Heart Involvement

True cardiac involvement manifests as atrioventricular conduction disturbances, tachyarrhythmias, myocarditis, and rarely heart failure and is more common in patients with ASyS and IMNM. In cases of active myositis, cardiac enzymes such as cardiac troponins may be elevated [14]. Sudden death may be caused by supraventricular tachyarrhythmias [15].

### 1.6. Gastrointestinal Involvement

Dysphagia is the most common GI symptom in patients with IIM and is associated with increased morbidity, due to its complications like aspiration pneumonia and weight loss resulting in malnutrition [10,11]. Sometimes intestinal mucosal inflammation occurs, with a high mortality rate [2].

### 1.7. Treatment

IIM treatment includes induction and maintenance therapy [10]. GCSs remain the main pillar of both, despite their systemic side effects. Prednisone is usually favoured as the initial approach, administered at 1 mg/kg body weight daily for 4 weeks, then tapered gradually to 5–20 mg per week. A lower prednisone dose may be sufficient in mildly symptomatic IIM patients. Methylprednisolone in doses of 500–1000 mg/day or dexamethasone pulse therapy at 40 mg/day for 3 consecutive days, with 28-day intervals in 3–6 cycles, can be as effective as daily prednisone [10,11].

Induction with GCS-sparing therapies includes IVIG (for dysphagia), MTX, AZA, MMF, calcineurin inhibitors, JAK inhibitors, RTX, and CYC. An ineffective induction should be modified by adding another induction agent; for example, JAK inhibitors, primarily brepocitinib and tofacitinib, have shown promising results but require further research [10,11]. New therapies, such as CD19+ CAR-T-cell therapy, should also be considered [10]. Abatacept in a randomised controlled trial does not seem effective, yet it can be beneficial in patients with PM and IMNM [10,11]. RTX is administered to patients refractory to GCSs and other GCS-sparing agents or those at high risk of developing severe ILD. Complete response is observed in 45% of such patients [11].

Maintenance with GCS-sparing therapies includes IVIG, MTX, AZA, RTX, MMF, and calcineurin inhibitors. IVIG and RTX seem to be beneficial especially in refractory cases. Ineffective maintenance should be modified by adding another maintenance agent [11].

Management of ILD-IIM should be tailored to the disease’s clinical manifestation. Induction treatment should involve GCSs and other immunosuppressive agents (MMF, AZA, cyclosporine [CsA], tacrolimus [TAC], or CYC). Worse-prognosis patients may require GCS treatment combined with RTX [12].

### 1.8. Dermatomyositis

DM is characterised by moderate myopathy, cutaneous rashes, and progressive muscular weakness, as well as extramuscular symptoms such as ILD and cardiovascular disease. In 20% of cases, DM is a mask of cancer, which should be excluded during the first 2 years of the patient follow-up. Various malignancies have been observed, like ovarian, lung, colorectal, and pancreatic cancer, as well as non-Hodgkin lymphoma [1,14]. Pathognomonic skin findings include Gottron’s papules and Gottron’s sign. Heliotrope rash is highly characteristic and can be used to monitor disease activity [14].

Serological markers include anti-Mi-2, anti-TIF1γ, anti-MDA5, and anti-NXP2. Anti-TIF-1 and anti-NXP-2 antibodies have also been shown to correlate with tumour growth [1,14]. Anti-Mi-2 correlates with a favourable prognosis [14]. Anti-MDA-5 can result in cutaneous ulcers, severe non-scarring alopecia, painful erythematous macules on the palms or the palmar surfaces of interphalangeal joints, mechanic’s hands, arthritis, and ILD [11].

Cutaneous calcinosis localised on the limbs and trunk correlates with the progression of the disease and is associated with presence of anti-NXP2 antibodies, and its complications include infection, ulceration, and limited joint mobility [11,14]. Known risk factors for developing DM include UV radiation exposure, the HLA-8.1 allele, and non-HLA alleles *PLCL1* and *BLK* [1].

### 1.9. Clinically Amyopathic Dermatomyositis

CADM is defined by cutaneous features of DM without muscle involvement with a high risk of ILD [3,11,16]. Anti-MDA5 CADM is the most common, especially in Asian populations, where it accounts for up to 35% of DM cases (compared to 7–15% in the European population) [16]. Skin symptoms that differ from other DM cases include a dark violaceous Gottron’s sign, reversion of Gottron’s papules to the palmar side of the hand, and the presence of ulcerations [11,16]. Due to RP-ILD risk, these patients require urgent, aggressive treatment [16].

### 1.10. Polymyositis

PM is a very rare condition that presents as isolated myositis [5,11]. Histopathological changes are diverse and, foremost, crucial for differentiating from IBM [5,10]. The most significant finding is the absence of rimmed vacuoles and amyloid deposits. No MSA or MAA antibodies are specific for PM, and the detection of many of these autoantibodies has allowed for the correct re-diagnosis of patients previously diagnosed with PM [7]. Known risk factors for developing PM include hepatitis B infection, the HLA-8.1 allele, and non-HLA alleles *PTPN22*. HIV and HTLV-1 infections can trigger PM [1].

### 1.11. Inclusion Body Myositis

IBM is characterised by the gradual progression of muscle weakness, most evident asymmetrically in the knee extensors (quadriceps femoris) and deep finger flexors [17,18]. This phenotype is characteristic but not pathognomic [17,18]. Anti-cN-1A have been associated with IBM. ILD has not been observed in patients with IBM [18]. The histopathological changes are quite specific, like rimmed vacuoles, amyloid deposits, and endomysial inflammation. Dysphagia is also common, especially in women, as is facial paresis [11,17,18]. IBM is relatively resistant to standard immunosuppressive therapy and GCSs [10,18]. Management focuses on maintaining function through physical therapy, exercise, and occupational therapy.

### 1.12. Immune-Mediated Necrotising Myopathy

IMNM manifests clinically in three phenotypes. Anti-HMGCR autoantibodies correlate with severe muscle involvement, without skin, lung, or joint symptoms [13,18]. It is linked to statin use in patients with the HLA-DRB1:11*01 allele; however, most patients do not have history of statin intake [14]. Anti-SRP are associated with severe muscle symptoms, cardiac involvement, ILD, and dysphagia [11].

### 1.13. Anti-Synthetase Syndrome

ASyS is identified by autoantibodies against aminoacyl-tRNA synthetases: anti-Jo-1, anti-PL-7, anti-PL-12, anti-EJ, anti-OJ, anti-KS, anti-Zo, and anti-Ha. Clinical presentation includes arthritis, myositis, and ILD in 20% of cases [8,12]. Joint involvement usually manifests as symmetrical, small-joint, non-erosive polyarthritis [8,10]. Other frequent symptoms include Raynaud phenomenon, fever, and mechanic’s hands [3]. About 20% of patients experience skin involvement, including mechanic’s hands, hiker’s feet, Gottron’s sign or papules, heliotrope rash, shawl sign, and calcinosis [8,11]. The phenotype without myositis but more common ILD is associated with non-anti-Jo-1 antibodies. Anti-Jo-1 is associated with better prognosis, in contrast to anti-PL-7 or anti-PL-12, which correlate with higher incidence of ILD and mortality [11].

### 1.14. Juvenile Dermatomyositis

JDM is a very rare immune-mediated IIM disease with an incidence of 2–4 cases per million [19,20].

JDM can be represented by a few autoantibodies [19]. Anti-Mi2 correlates with less severe disease courses, lower mortality, but muscle involvement is severe. This phenotype responds well to standard treatment [19,20,21]. Anti-NXP2 and anti-TIF-1γ correlate with more severe disease courses and lower remission rates. Anti-TIF-1γ correlates with milder muscle involvement and more severe skin symptoms, but, in contrast to adult DM patients, this phenotype in the paediatric population does not correlate with malignancy [19,21]. The anti-NXP2 phenotype is characterised by muscle and gastrointestinal involvement, as well as calcinosis. Anti-MDA5 positivity is associated with persistent disease and less severe muscle involvement [16]. This phenotype, especially when co-existing with anti-Ro52, is at greater risk of developing ILD [20]. The amyopathic phenotype is very rare and is associated with anti-MDA5, but rarely with anti-TIF-1γ. Sparse muscle involvement should prompt anti-MDA5 investigations to identify patients at risk of developing ILD [19,21]. The immune-mediated necrotising myopathy phenotype often presents with both muscle and skin involvement (manifesting as atypical rashes). It correlates with anti-SRP and anti-HMGCR, both of which are associated with severe muscle symptoms, elevated creatinine kinase levels, and treatment resistance [20]. Use of statins is not associated with the development of anti-HMGCR autoantibodies in children [19,21]. The anti-synthetase phenotype is rare, occurring in fewer than 5% of paediatric patients. It is associated with older age at diagnosis (median of 14 years, compared with 6,5 years for JDM overall). Anti-Jo-1 is the most common [22]. Clinical manifestations include mechanic’s hands, arthritis, Raynaud’s phenomenon, fever, ILD, and myositis [19,21]. GCS therapy has greatly reduced JDM mortality [20].

## 2. Methods and Materials

A single-centre, retrospective cohort study was conducted at the Rheumatology Clinic of the National Institute of Geriatrics, Rheumatology and Rehabilitation in Warsaw, Poland. The study included 35 patients with IIM hospitalised between 1 January 2022, and 31 December 2025. Selection criteria included IIM diagnosis and hospitalisation in our centre. All patients fulfilled these selection criteria. Diagnosis was confirmed according to the 2017 EULAR classification criteria [4] and supplemented by Gianni et al. [6]. No case of overlap myositis was observed.

### 2.1. Data Collection

Data collection from medical records included the following:

— Demographics: age, sex, body mass index (BMI), death;

— Disease characteristics: disease duration, IIM type;

— Extramuscular involvement: comprehensive data were collected on any cutaneous, cardiac, articular, and oesophageal manifestations. All symptoms reported by the patient or signs observed by the physician were taken into account. The presence of calcinosis was noted.

— Co-existence of Sjögren syndrome diagnosed in accordance with 2016 ACR-EUCLAR classification criteria [22].

— Biopsy performance and its findings.

— Serology: MSAs and ANAs positivity and type.

— Treatment interventions: use of GCSs, CYC, CsA, MTX, MMF, AZA, JAK inhibitors, RTX, anifrolumab, nintedanib, or sirolimus.

In all cases, medical records provided all needed data. No missing data were found.

### 2.2. Data Analysis

Categorical variables were coded binarily (1 = present, 0 = absent; sex: 3 = male, and 2 = female). Descriptive statistics were employed: continuous variables are presented as median and range, and categorical variables as counts and percentages (*n*, %). The cohort consisted of 35 patients (24 women and 11 men): 10 DM, 1 PM, 2 IMNM, 1 IBM, 16 ASS, 4 JDM, and 1 CADM cases. Due to the sample size, all analyses are descriptive.

### 2.3. Ethical Considerations

The study protocol adhered to the principles of the Declaration of Helsinki and Good Clinical Practice. Approval was granted by the institutional bioethics committee (nr KBT-2/5/2026 dated 26 February 2026). Informed consent was obtained from all participants.

## 3. Results

The cohort included 11 men and 24 women. The mean age was 50.79 years (median 49, range 19–75). The mean BMI was 26.0 kg/m^2^. The mean disease duration was 9.6 (median 6.0, range 1–43). JDM cases included in this study, diagnosed in childhood and hospitalised as adults in our centre, account for the higher end of this range.

Demographic and clinical data are presented in Table 1.

### 3.1. Extramuscular Involvement

Skin was most commonly affected, aside from the muscular system. Notably, 93.8% of ASyS cases exhibited cutaneous manifestations, compared with only 50% of DM patients.

In total, 60% of patients suffered from ILD, most commonly of the NSIP phenotype; 93.8% of ASyS patients were affected, consistent with the literature [1,8,12].

Cardiac and articular involvement were most common among ASyS patients.

Half of the dysphagia cases were attributed to DM, and the other half consisted of two ASyS and one IBM case, the last of which is quite uncharacteristic [11].

Raynaud’s phenomenon was observed in three DM and four ASyS patients.

Notably, six patients with ASyS, one patient with DM, and one patient with IMNM were also diagnosed with co-existing Sjögren syndrome disease.

Calcinosis was confirmed in four cases, three of which were JDM, consistent with literature reports [19,20].

Co-occurrence of malignancy was noted in two DM cases.

### 3.2. Serology

Serology markers are presented in Table 2.

Thirty patients had detectable MSAs. Three patients were MSA seronegative. One patient was negative for both MAAs and MSAs.

Twenty patients had detectable MAAs; nineteen had co-occurring anti-Ro52 and an MSA, and one had an unspecified ANA.

Anti-Jo-1 was the most common MSA, occurring in thirteen out of sixteen ASyS cases and one DM.

In one of the IMNM patients, anti-SRP was confirmed, and in the other, anti-HMGCR, consistent with literature reports [10,17].

Malignancy co-existed in one case with anti-TIF1-γ and in one with anti-SAE.

Multiple MSAs were found in only two patients, in whom anti-HMGCR co-occurred with either anti-PL-7 or anti-NXP-2.

### 3.3. Histopathological Findings in Muscle Biopsy Specimens

Muscle pathology was performed in sixteen patients, and its characteristics are presented in Table 3.

Two pathognomonic features of DM were observed. Perifascicular atrophy was observed in three out of six DM samples and two out of two JDM cases, and perivascular lymphocyte infiltration occurred in three out of six DM specimens and in one JDM case. Such findings in JDM patients suggest a DM phenotype in the clinical course of disease. Necrosis of muscle fibres, pathognomonic of IMNM, was confirmed in two of two cases. It was also found in one DM and one JDM sample, indicating a very aggressive inflammatory response.

Characteristic for IBM dystrophic changes were present in the one confirmed case. Membrane attack complex (MAC) C5-b9 was found in one of two IMNM cases, demonstrating complement activation present in the disease’s course.

### 3.4. Treatment Interventions

Pharmacotherapy is presented in Table 4.

Induction treatment included GCSs in high doses (500–1000 mg i.v.) in all patients. Additionally, CYC was added in 10 cases, MMF in 18, and MTX in 6 (all within the DM spectrum). If possible, after induction, GCSs were discontinued in favour of monotherapy or a combination of other immunosuppressive agents. However, at least at the beginning of maintenance therapy, GCSs were administered in small doses (5–20 mg/week) to all patients (to gradually taper off the dose).

One patient with a malignancy co-morbidity after major adverse effects associated with GCS use was disqualified from further immunosuppressive therapies. As salvage therapy, IVIG was successfully administered. Nine other patients received IVIG ordinarily as a maintenance therapy.

Maintenance therapy was often modified during follow-up based on clinical status. If remission was not achieved with traditional therapies, novel therapies were administered. In some cases, novel therapies were first-line maintenance therapy. Twelve patients received rituximab. Five patients received JAK inhibitors. Those included two DM, two JDM, and one ASyS case. Addition of nintedanib for progressive ILD was necessary in four patients, two of whom suffered from ASyS. Anifrolumab was administered in one DM and one JDM case. Only one patient, diagnosed with IBM, received sirolimus.

The safety profile and disease control were acceptable.

## 4. Discussion

This single-centre study from a Polish highly specialised clinic demonstrates a wide variety of IIM diagnoses and their phenotypes. Certain observations can be made about the cohort. ASyS was the most common IIM diagnosis, with anti-Jo-1 being the most frequent MSA detected, consistent with the literature. In our study, the frequency of a single MAA in the cohort is noteworthy: anti-Ro52 antibody. It has been proven to be one of the most common MAAs in patients with IIM [7].

Interestingly, primary Sjögren’s syndrome was diagnosed in six ASyS cases, one DM case, and one IMNM case. The available literature on this remains limited, consisting mainly of small case reports and reviews; no association with other connective diseases is even rarer [23]. Co-occurrence of IIM and Sjögren’s is increasingly recognised as a distinct clinical phenotype. Konen et al. reported the overlap mostly in PM and DM cases, as well as IBM [24]. In the case series and literature review by Migkos et al. the overlap was predominantly observed in PM and IBM, with DM being relatively uncommon [25]. Neither observed the co-occurrence between Sjögren’s and ASyS or IMNM [24,25]. In this context, the relatively high frequency of Sjögren’s overlap with ASyS in our study population is noteworthy and contrasts with the existing literature. This discrepancy may reflect differences in patient selection, referral bias inherent to a highly specialised centre or potentially a new association that warrants further investigation. The high prevalence of anti-Ro52 in the cohort may be a factor, as it has been independently and in association with IIM linked to Sjögren’s syndrome [25,26]. This requires further research, as such a distinctive group of patients may benefit from certain treatment regimens.

A similar case series was reported by Tharwat et al. [9]. They described a cohort of 99 patients, mostly women. The most common diagnosis was PM, followed by DM, and overlap myositis accounted for 19.2% of cases. This differs from our study population. Muscle biopsy was performed in only four patients, whereas MRI was performed in just over half of the study population. This contrasts with our study and reflects differences in diagnostic approaches as well as the higher prevalence of PM in this cohort. The use of novel therapies was also reported, with rituximab being the most frequently administered drug, which is consistent with our observations. Notably, a wider range of both conventional and novel therapies was described compared to our centre, including leflunomide, hydroxychloroquine, sulfasalazine, colchicine, and anti-TNF agents [9].

In our study, all patients received intermittent GCSs; this remains the mainstay of IIM treatment. Adverse effects associated with long-term GCS use necessitate the search for new therapies. Four novel therapies were used: rituximab, JAK inhibitors, anifrolumab, and sirolimus.

Because IIMs are rare diseases, conducting randomized, controlled trials is difficult. The primary sources of data on the effectiveness of new drugs are primarily case reports and case series of successful use of various therapies.

Rituximab, either as monotherapy or in combination with mycophenolate mofetil, has been shown to be effective in IIM. Its efficacy in refractory cases has also been observed, yet randomised controlled trials failed to reach their primary endpoints [27,28].

JAK inhibitors have been recognised as a novel treatment for IIM. Two of the treated patients were suffering from DM, another two from JDM, and one from ASyS. Their effective use is supported by the literature [29,30].

To our knowledge, anifrolumab has been reported to be effective in IIM treatment only a few times—four in JDM [31] and three in DM [32]. In our cohort, it was administered with good results in one JDM and one DM patient. This treatment requires further observation in ongoing clinical trials; nevertheless, this report, consistent with the limited data available, appears promising.

There is a strong need for an effective IBM treatment due to the limited effectiveness of traditional therapies. It was reported casuistically and in some clinical trials to improve the symptoms [33,34,35]. Clonal expansion and muscle infiltration by CD8+ T-cells plays a crucial role in pathogenesis of IBM. They are terminally differentiated and display high cytotoxicity. A higher proportion of Th1 is also observed. Sirolimus depletes effector T-cells and preserves Treg by inhibiting mammalian target of rapamycin (mTOR), which is vital to many proliferation and regulatory cell pathways. It also prompts autophagy, which is also disrupted in IBM [33,35]. The one patient from our cohort with IBM was treated with Sirolimus with good results.

This study confirms the rarity of true PM cases—only one of thirty-five patients in a very highly specialised centre fulfilled the EUCLAR and Gianni et al. criteria over three years.

Several limitations of this study should be acknowledged. First, IIM is a very rare disease, and even in our highly specialised centre, our sample size remains very small. Consequently, all analyses are limited to descriptive methods, which are inherently prone to bias and are subject to error. Second, the cohort consists solely of patients requiring repeated hospitalisations and excludes those managed exclusively in the outpatient clinic. These selection criteria may result in overrepresentation of more advanced, severe, or refractory cases. Third, referral to a highly specialised centre itself increases the likelihood of including patients with atypical or more severe diseases courses, further contributing to this bias. As such, the drawing of meaningful conclusions is limited.

## 5. Conclusions

This Polish single-centre cohort shows novel therapies (rituximab, JAK inhibitors, anifrolumab, sirolimus) for IIM. Sirolimus was effectively administered to an IBM patient. The results of treatment with JAK inhibitors are very interesting and promising. Nintedanib was effective in ILD management in our patients.

This study shows the high prevalence of ASyS patients and of patients with co-occurring Sjögren disease. Co-occurrence of SD in ASyS patients requires further research.

The need for an interdisciplinary care provision for IIM patients is reflected. Lung involvement, common among ASyS patients, requires early screening, appropriate pulmonary management, and experienced radiological follow-up.

## Figures and Tables

**Table 1:** Demographic characteristics, organ involvement, and clinical features (N = 35).

	Overall (N = 35)	Women (n = 24)	Men (n = 11)	DM (n = 10)	ASS (n = 16)	JDM (n = 4)	PM (n = 1)	IMNM (n = 2)	IBM (n = 1)	CADM (n = 1)
A. Demographics										
Female	24 (68.6%)	24 (100.0%)	0 (0%)	9 (90.0%)	9 (56.2%)	3 (75.0%)	0 (0%)	2 (100.0%)	1 (100.0%)	0 (0%)
Male	11 (31.4%)	0 (0%)	11 (100.0%)	1 (10.0%)	7 (43.8%)	1 (25.0%)	1 (100.0%)	0 (0%)	0 (0%)	1 (100.0%)
	mean/median (range)	mean/median (range)	mean/median (range)	mean/median (range)	mean/median (range)	mean/median (range)	mean/median (range)	mean/median (range)	mean/median (range)	mean/median (range)
Age, years	50.7/49.0 (19–75)	50.1/47.5 (19–75)	51.9/49.0 (21–75)	47.9/41.5 (29–75)	53.2/49.0 (33–75)	40.5/45.0 (21–51)	61.0/61.0 (61–61)	45.0/45.0 (19–71)	62.0/62.0 (62–62)	68.0/68.0 (68–68)
BMI, kg/m^2^	26.0/25.3 (16–41)	25.7/24.8 (16–41)	26.7/27.2 (17–34)	24.2/24.8 (20–28)	28.9/27.2 (19–41)	22.3/21.9 (17–28)	25.7/25.7 (26–26)	22.6/22.6 (16–29)	18.2/18.2 (18–18)	27.2/27.2 (27–27)
Disease duration, years	9.6/6.0 (1–43)	11.2/8.0 (1–43)	6.2/4.0 (2–13)	3.7/2.5 (1–12)	8.2/8.0 (2–17)	31.8/35.5 (13–43)	2.0/2.0 (2–2)	12.0/12.0 (8–16)	11.0/11.0 (11–11)	4.0/4.0 (4–4)
B. Interstitial lung disease (ILD)										
Any ILD	21 (60.0%)	14 (58.3%)	7 (63.6%)	5 (50.0%)	15 (93.8%)	0 (0%)	0 (0%)	1 (50.0%)	0 (0%)	0 (0%)
NSIP	16 (45.7%)	10 (41.7%)	6 (54.5%)	4 (40.0%)	11 (68.8%)	0 (0%)	0 (0%)	1 (50.0%)	0 (0%)	0 (0%)
UIP	2 (5.7%)	1 (4.2%)	1 (9.1%)	0 (0%)	2 (12.5%)	0 (0%)	0 (0%)	0 (0%)	0 (0%)	0 (0%)
OP	4 (11.4%)	2 (8.3%)	2 (18.2%)	1 (10.0%)	3 (18.8%)	0 (0%)	0 (0%)	0 (0%)	0 (0%)	0 (0%)
LIP	1 (2.9%)	1 (4.2%)	0 (0%)	0 (0%)	1 (6.2%)	0 (0%)	0 (0%)	0 (0%)	0 (0%)	0 (0%)
Unspecified	0 (0%)	0 (0%)	0 (0%)	0 (0%)	0 (0%)	0 (0%)	0 (0%)	0 (0%)	0 (0%)	0 (0%)
C. Other organ and system involvement										
Skin involvement	28 (80.0%)	19 (79.2%)	9 (81.8%)	10 (100.0%)	13 (81.2%)	4 (100.0%)	0 (0%)	0 (0%)	0 (0%)	1 (100.0%)
Cardiac involvement	10 (28.6%)	7 (29.2%)	3 (27.3%)	2 (20.0%)	7 (43.8%)	1 (25.0%)	0 (0%)	0 (0%)	0 (0%)	0 (0%)
Joint involvement	9 (25.7%)	6 (25.0%)	3 (27.3%)	3 (30.0%)	6 (37.5%)	0 (0%)	0 (0%)	0 (0%)	0 (0%)	0 (0%)
Oesophageal dysmotility	8 (22.9%)	6 (25.0%)	2 (18.2%)	4 (40.0%)	2 (12.5%)	0 (0%)	0 (0%)	0 (0%)	1 (100.0%)	1 (100.0%)
Raynaud’s phenomenon	7 (20.0%)	4 (16.7%)	3 (27.3%)	3 (30.0%)	4 (25.0%)	0 (0%)	0 (0%)	0 (0%)	0 (0%)	0 (0%)
Calcinosis cutis	4 (11.4%)	3 (12.5%)	1 (9.1%)	1 (10.0%)	0 (0%)	3 (75.0%)	0 (0%)	0 (0%)	0 (0%)	0 (0%)
Co-occurring Sjogren’s syndrome	8 (22.9%)	7 (29.2%)	1 (9.1%)	1 (10.0%)	6 (37.5%)	0 (0%)	0 (0%)	1 (50.0%)	0 (0%)	0 (0%)
Co-occurrence of malignancy	2 (5.7%)	2 (8.3%)	0 (0%)	2 (20.0%)	0 (0%)	0 (0%)	0 (0%)	0 (0%)	0 (0%)	0 (0%)

Values: mean/median (range) for continuous variables; n (%) for categorical. DM = dermatomyositis; ASS = anti-synthetase syndrome; JDM = juvenile DM; PM = polymyositis; IMNM = immune-mediated necrotising myopathy; IBM = inclusion body myositis; CADM = clinically amyopathic DM. ILD = interstitial lung disease; NSIP = non-specific interstitial pneumonia; UIP = usual interstitial pneumonia; OP = organising pneumonia; LIP = lymphoid interstitial pneumonia. Malignancy: found in two DM patients (one with anti-TIF1-γ and one with anti-SAE). ILD subtypes are not mutually exclusive.

**Table 2:** Myositis-specific and myositis-associated antibodies (MSAs/MAAs) in inflammatory myopathies (N = 35).

	Overall (N = 35)	DM (n = 10)	ASS (n = 16)	JDM (n = 4)	PM (n = 1)	IMNM (n = 2)	IBM (n = 1)	CADM (n = 1)
**Myositis-specific antibodies (MSAs)**								
Anti-Jo-1	14 (40.0%)	1 (10.0%)	13 (81.2%)	0 (0%)	0 (0%)	0 (0%)	0 (0%)	0 (0%)
Anti-PM-Scl	3 (8.6%)	3 (30.0%)	0 (0%)	0 (0%)	0 (0%)	0 (0%)	0 (0%)	0 (0%)
Anti-TIF1-γ §	2 (5.7%)	1 (10.0%)	0 (0%)	1 (25.0%)	0 (0%)	0 (0%)	0 (0%)	0 (0%)
Anti-PL-12	2 (5.7%)	1 (10.0%)	1 (6.2%)	0 (0%)	0 (0%)	0 (0%)	0 (0%)	0 (0%)
Anti-SRP	1 (2.9%)	0 (0%)	0 (0%)	0 (0%)	0 (0%)	1 (50.0%)	0 (0%)	0 (0%)
Anti-Scl-70	1 (2.9%)	0 (0%)	1 (6.2%)	0 (0%)	0 (0%)	0 (0%)	0 (0%)	0 (0%)
Anti-Mi-2	1 (2.9%)	1 (10.0%)	0 (0%)	0 (0%)	0 (0%)	0 (0%)	0 (0%)	0 (0%)
Anti-HMGCR †‡	3 (8.6%)	1 (10.0%)	0 (0%)	0 (0%)	1 (100.0%)	1 (50.0%)	0 (0%)	0 (0%)
Anti-PL-7 †	2 (5.7%)	0 (0%)	1 (6.2%)	0 (0%)	1 (100.0%)	0 (0%)	0 (0%)	0 (0%)
Anti-NXP2 ‡	2 (5.7%)	2 (20.0%)	0 (0%)	0 (0%)	0 (0%)	0 (0%)	0 (0%)	0 (0%)
Anti-SAE ¶	1 (2.9%)	1 (10.0%)	0 (0%)	0 (0%)	0 (0%)	0 (0%)	0 (0%)	0 (0%)
**No MSA detected**	**4 (11.4%)**	0 (0%)	0 (0%)	2 (50.0%)	0 (0%)	0 (0%)	1 (100.0%)	1 (100.0%)
**Myositis-associated antibodies (MAAs) and ANAs**								
Anti-Ro/SSA	19 (54.3%)	4 (40.0%)	14 (87.5%)	0 (0%)	0 (0%)	1 (50.0%)	0 (0%)	0 (0%)
ANA positive (specificity not determined)	1 (2.9%)	0 (0%)	0 (0%)	1 (25.0%)	0 (0%)	0 (0%)	0 (0%)	0 (0%)
**Co-occurring MSAs (n = 2 patients)**								
Anti-PL-7 + anti-HMGCR †	1 (2.9%)	-	-	-	1 (100.0%)	-	-	-
Anti-NXP2 + anti-HMGCR ‡	1 (2.9%)	1 (10.0%)	-	-	-	-	-	-

n (%) with respect to group size. MSAs = myositis-specific antibodies; MAAs = myositis-associated antibodies; ANAs = antinuclear antibodies; DM = dermatomyositis; ASS = anti-synthetase syndrome; JDM = juvenile DM; PM = polymyositis; IMNM = immune-mediated necrotising myopathy; IBM = inclusion body myositis; CADM = clinically amyopathic DM. † Anti-PL-7 co-occurring with anti-HMGCR (PM, 1 patient). ‡ Anti-NXP2 co-occurring with anti-HMGCR (DM, 1 patient). § Anti-TIF1-γ: 1/2 patients with co-occurring malignancy. ¶ Anti-SAE: associated with thyroid carcinoma (1 patient). Data presented without patient identifiers.

**Table 3:** Histopathological findings in muscle biopsies (n biopsied = 16/35).

	Total Biopsied (n = 16)	DM (n = 6)	ASS (n = 4)	JDM (n = 2)	PM (n = 1)	IMNM (n = 2)	IBM (n = 1)
Necrosis of muscle fibres	4 (25.0%)	1 (16.7%)	0 (0%)	1 (50.0%)	0 (0%)	2 (100.0%)	0 (0%)
Perifascicular muscle fibre atrophy	5 (31.2%)	3 (50.0%)	2 (50.0%)	0 (0%)	0 (0%)	0 (0%)	0 (0%)
Perivascular lymphocyte infiltration	4 (25.0%)	4 (66.7%)	0 (0%)	0 (0%)	0 (0%)	0 (0%)	0 (0%)
Dispersed lymphocyte infiltration	8 (50.0%)	1 (16.7%)	3 (75.0%)	1 (50.0%)	0 (0%)	2 (100.0%)	1 (100.0%)
MAC5-B9 complement deposition	1 (6.2%)	0 (0%)	0 (0%)	0 (0%)	0 (0%)	1 (50.0%)	0 (0%)
Dystrophic changes	1 (6.2%)	0 (0%)	0 (0%)	0 (0%)	0 (0%)	0 (0%)	1 (100.0%)

n (%) with respect to biopsied patients per group. Biopsies not performed in CADM (n = 0). Findings are not mutually exclusive. DM = dermatomyositis; ASS = anti-synthetase syndrome; JDM = juvenile DM; PM = polymyositis; IMNM = immune-mediated necrotising myopathy; IBM = inclusion body myositis. MAC5-B9 = membrane attack complex component 5b-9 (marker of terminal complement activation).

**Table 4:** Treatment characteristics of patients with inflammatory myopathies (N = 35): induction and maintenance therapy.

	Overall (N = 35)		DM (n = 10)		ASS (n = 16)		JDM (n = 4)		PM (n = 1)		IMNM (n = 2)		IBM (n = 1)		CADM (n = 1)	
Treatment	Ind.	Maint.	Ind.	Maint.	Ind.	Maint.	Ind.	Maint.	Ind.	Maint.	Ind.	Maint.	Ind.	Maint.	Ind.	Maint.
1. Conventional immunosuppressive therapies																
Corticosteroids (GCSs)—all patients	35/35 (100%)	35/35 (100%)	10/10 (100%)	10/10 (100%)	16/16 (100%)	16/16 (100%)	4/4 (100%)	4/4 (100%)	1/1 (100%)	1/1 (100%)	2/2 (100%)	2/2 (100%)	1/1 (100%)	1/1 (100%)	1/1 (100%)	1/1 (100%)
Cyclophosphamide (CYC)	10 (28.6%)	0 (0%)	1 (10.0%)	0 (0%)	7 (43.8%)	0 (0%)	1 (20.0%)	0 (0%)	0 (0%)	0 (0%)	1 (100.0%)	0 (0%)	0 (0%)	0 (0%)	0 (0%)	0 (0%)
Methotrexate (MTX)	6 (17.1%)	7 (20.0%)	3 (30.0%)	4 (40.0%)	0 (0%)	1 (6.2%)	1 (20.0%)	0 (0%)	1 (100.0%)	1 (100.0%)	0 (0%)	0 (0%)	0 (0%)	0 (0%)	1 (100.0%)	1 (100.0%)
Mycophenolate mofetil (MMF)	18 (51.4%)	11 (31.4%)	5 (50.0%)	1 (10.0%)	9 (56.2%)	9 (56.2%)	3 (60.0%)	1 (20.0%)	0 (0%)	0 (0%)	0 (0%)	0 (0%)	1 (100.0%)	0 (0%)	0 (0%)	0 (0%)
Intravenous immunoglobulins (IVIG)	—	10 (28.6%)	—	4 (40.0%)	—	3 (18.8%)	—	2 (50.0%)	—	0 (0%)	—	1 (50.0%)	—	0 (0%)	—	0 (0%)
Cyclosporine A (CSA)	—	6 (17.1%)	—	3 (30.0%)	—	2 (12.5%)	—	1 (25.0%)	—	0 (0%)	—	0 (0%)	—	0 (0%)	—	0 (0%)
Azathioprine (AZA)	—	15 (42.9%)	—	4 (40.0%)	—	8 (50.0%)	—	1 (25.0%)	—	0 (0%)	—	1 (50.0%)	—	1 (100.0%)	—	0 (0%)
2. Novel/targeted therapies																
Rituximab (RTX)	0 (0%)	12 (34.3%)	0 (0%)	2 (20.0%)	0 (0%)	7 (43.8%)	0 (0%)	1 (25.0%)	0 (0%)	0 (0%)	0 (0%)	2 (100.0%)	0 (0%)	0 (0%)	0 (0%)	0 (0%)
JAK inhibitors	0 (0%)	5 (14.3%)	0 (0%)	2 (20.0%)	0 (0%)	1 (6.2%)	0 (0%)	2 (50.0%)	0 (0%)	0 (0%)	0 (0%)	0 (0%)	0 (0%)	0 (0%)	0 (0%)	0 (0%)
Nintedanib §	0 (0%)	4 (11.4%)	0 (0%)	1 (10.0%)	0 (0%)	2 (12.5%)	0 (0%)	0 (0%)	0 (0%)	0 (0%)	0 (0%)	1 (50.0%)	0 (0%)	0 (0%)	0 (0%)	0 (0%)
Anifrolumab	0 (0%)	2 (5.7%)	0 (0%)	1 (10.0%)	0 (0%)	0 (0%)	0 (0%)	1 (25.0%)	0 (0%)	0 (0%)	0 (0%)	0 (0%)	0 (0%)	0 (0%)	0 (0%)	0 (0%)
Sirolimus *	0 (0%)	1 (2.9%)	0 (0%)	0 (0%)	0 (0%)	0 (0%)	0 (0%)	0 (0%)	0 (0%)	0 (0%)	0 (0%)	0 (0%)	0 (0%)	1 (100.0%)	0 (0%)	0 (0%)
Any novel therapy	0 (0%)	20 (57.1%)	0 (0%)	6 (60.0%)	0 (0%)	8 (50.0%)	0 (0%)	3 (75.0%)	0 (0%)	0 (0%)	0 (0%)	2 (100.0%)	0 (0%)	1 (100.0%)	0 (0%)	0 (0%)

Ind. = induction therapy; Maint. = maintenance therapy * Sirolimus: IBM only (n = 1). § Nintedanib used in progressive ILD alongside other therapies. GCSs = glucocorticosteroids; CYC = cyclophosphamide; MTX = methotrexate; MMF = mycophenolate mofetil; IVIG = intravenous immunoglobulins; CSA = cyclosporine A; AZA = azathioprine; RTX = rituximab; JAK = Janus kinase inhibitors (tofacitinib/baricitinib); DM = dermatomyositis; ASS = anti-synthetase syndrome; JDM = juvenile DM; PM = polymyositis; IMNM = immune-mediated necrotising myopathy; IBM = inclusion body myositis; CADM = clinically amyopathic DM.

## Data Availability

Data are available upon reasonable request according to ICMJE requirements. Data shared: All the individual participant data were collected during the trial, after deidentification. Available documents: study protocol, statistical analysis, analytic code. When will data be available: beginning three months and ending two years following the article’s publication. With whom: researchers who provide a methodologically sound proposal. For what type of analyses: to achieve the aim of the approved proposal. By what mechanism will data be made available: The proposal should be directed to julia-domanska03@wp.pl.

## Abbreviations

DM	dermatomyositis
PM	polymyositis
AsyS	antisynthetase syndrome
IMNM	immune-mediated necrotising myopathy
IBM	inclusion body myositis
CADM	clinically amyopathic dermatomyositis
OM	overlap myositis
CAM	cancer-associated myositis
PLCL1	phospholipase C-like 1
BLK	B lymphoid tyrosine kinase
GCSs	glucocoritcoids
MTX	methotrexate
MMF	mycophenolate mofetil
AZA	azathioprine
RTX	rituximab
CSA	cyclosporine A
IVIG	intravenous immunoglobulins
CYC	cyclophosphamide
MSAs	myositis-specific autoantibodies
MAAs	myositis-associated autoantibodies
anti-Jo-1	anti-histidyl tRNA synthetase antibody
anti-PL-7	anti-threonyl tRNA synthetase antibody
anti-PL-12	anti-alanyl tRNA synthetase antibody
anti-EJ	anti-glycyl tRNA synthetase antibody
anti-OJ	anti-isoleucyl tRNA synthetase antibody
anti-Zo	anti-phenylalanyl tRNA synthetase antibody
anti-KS	anti-asparaginyl tRNA synthetase antibody
anti-Ha	anti-tyrosyl tRNA synthetase antibody
anti-SRP	anti-signal recognition particle antibody
anti-Mi-2	anti-Mi-2 antibody
anti-MDA5	anti-melanoma differentiation-associated gene 5 antibody
anti-NXP2	anti-nuclear matrix protein 2 antibody
anti-TIF-1	anti-transcription intermediary factor 1 antibody
anti-SAE	anti-small ubiquitin-like modifier activating enzyme antibody
anti-PM/Scl	anti-PM/Scl antibody
anti-U1-RNP	anti-U1 ribonucleoprotein antibody
anti-Ro60	anti-Ro60 antibody
anti-Sm	anti-Smith antibody
anti-Ku	anti-Ku antibody
anti-La	anti-La (SSB) antibody
anti-U3	anti-U3 ribonucleoprotein antibody
anti-RNA Pol	anti-RNA polymerase antibody
anti-Topo	anti-topoisomerase I (Scl-70) antibody
ANA	antinuclear antibodies
AMA	antimitochondrial antibodies
ILD	interstitial lung disease
RP-ILD	rapidly progressive interstitial lung disease
